# Miscellaneous

**Published:** 1890-07

**Authors:** 


### MISCELLANEOUS.

### A GRATIFYING ACKNOWLEDGMENT.

NEW YORK, 23rd June, 1890.

HALL'S JOURNAL OF HEALTH.

GENTS:—Our Company deem it a business duty to certify to the advertising advantages of your publication. Having used no other medium than the Journal, and some of its exchanges in which it kindly arranged for the insertion of our advertisement, we ascribe our remarkable increase of patronage to it, and credit to it the success that has attended us from a small beginning, till now enabled to greatly enlarge our business and establish it in a better locality and upon a better basis. Enclosed find copy for an additional page advertisement.

Respectfully,

HERBA VITA REMEDY CO.,
218, 220, 222 Fulton St.

[We publish the above, not only as showing a full appreciation of the worth of our advertising pages, but for the sake of saying that we insert no ad. not deemed deserving of editorial endorsement, and that we will accept none at any price which we cannot cordially recommend to our readers. We are glad to give Herba Vita another page, as we personally know it to be really a remarkable remedy, and one which recommends itself to all who try it. Our readers can verify this by sending for free samples.—EDITOR.]

### CANDIES MADE OF WHITE CLAY.

In the matter of candies the conduct of parents is often extremely irrational. They would not themselves venture to use sweetmeats freely, and yet very many give them to their children with scarcely any restraint, and the sicklier and weaker the child, the more likely is it to be senselessly indulged. This fault would be grievous enough were the confectionery pure—what estimate can be put upon it in the light of the fact that very much of it on the market is made of spurious ingredients. It appears that not long ago a lawsuit led to a special investigation into the composition of certain kinds of candy. We are told that it was then developed that lozenges are made in enormous quantities of little else but white clay. The clay is compressed in moulds, and bound together with a little gum or gelatine, and immersed for a few moments in a "syrupy" bath containing the required flavor. This makes a very cheap form of confection, which is largely consumed by children. The adulteration or substitution is also carried on to some extent in the manufacture of caramels: but in the case of the cheap lozenges the proportions of clay used is the highest possible consistent with their maintenance of shape. The Portland Board of Trade Journal has stated that there is an annual importation of terra alba amounting to 6,000 tons, and that the only considerable use made of it is in the adulteration of the cheaper grades of candy.

### CURE FOR DRUNKENNESS.

An habitual drunkard in Sweden and Norway is treated as a criminal in this sense, that his inordinate love of strong drink renders him liable to imprisonment, and while in confinement it appears he is cured of his bad propensities on a plan which, though simple enough, is said to produce marvelous effects. From the day the confirmed drunkard is incarcerated no nourishment is served to him but bread and wine. The bread, however, it should be said, cannot be eaten apart from the wine, but is steeped in a bowl of it and left thus to soak an hour or more before the meal is served to the delinquent. The first day the habitual toper takes his food in this shape without the slightest repugnance ; the second day he finds it less agreeable to his palate, and very quickly he evinces a positive aversion to it. Generally, the doctor states, eight or ten days of this regimen is more than sufficient to make a man loathe the very sight of wine, and even refuse the prison dish set before him. This manner of curing drunken habits is said to succeed almost without exception, and men or women who have undergone the treatment not only rarely return to their evil ways, but from sheer disgust they frequently become total abstainers afterwards.

### THE POTATO.

The potato is one of the most important of cultivated plants, and in universal cultivations in temperate parts of the globe. It is a native of mountain districts of tropical and subtropical America, probably from Chilli to Mexico, but there is some question as to where it is really indigenous. Humboldt doubted if it had ever been found truly wild, but subsequent travelers of high scientific reputation express themselves thoroughly satisfied. Maize and potatoes are the two greatest gifts which America has given to the rest of the world.

The potato has been cultivated in America and its tubers used for food for a time long anterior to the discovery of America by Europeans. It seems to have been brought first to Europe by the Spaniards from the neighborhood of Quito in the sixth century. No more important event of this kind has ever taken place than the introduction of potato culture into Great Britain and other European countries. It was long called "Batatas" or sweet potato, which is the tuber or plant meant by English writers down to the middle of the seventeenth century. It appears to have been brought to "Ireland from Virginia, by Hawkins in 1565," "and to England by Sir Francis Drake in 1623."

Clouds and their Heights.—For practical purposes clouds are divided into four classes—cumulus, stratus, cirrus, and nimbus. Meteorologists, however, recognize many differences of form in each class. Abercrombie gives these ten principal varieties, with their mean height in summer at Upsola, Sweden : Cirrus (pure wispy cloud), 27,000 feet ; cirrostratus (thin, high, wispy, or striated, but at a low level), 15,000 feet ; cumulo-cirrus (fleecy cloud at low level), 12,000 feet ; strato-cumulus (extended lumpy cloud), 4,000 feet at base ; cumulo-nimbus (low rain cloud), 4,500 feet ; stratus (pure sheet cloud), 1,900 feet.

### PASTE FOR SCRAP-BOOKS.

Take equal parts of gum arabic and gum tragacanth, and dissolve with enough warm water so that it will be like thick gloss starch ; if, on trial, you find the paste too thick to spread nicely with a small brush, thin with more warm water, stirring until it is smooth again. A small amount of gum makes a lot of paste. Keep in a large mouthed bottle that can be covered tightly, and it will keep for months, yes, a year or more.

If it dries down hard or thick, soak it up with more warm water. A bottle of this stuff is very handy during fruit season for pasting labels on fruit cans or jars.

In making scrap-books, the slips of paper or pictures should be arranged on the page before commencing to paste any, as they will stick so quickly and so tightly that it is not easy to make changes.

A little experience with this paste will soon give you a scrap-book that will look much prettier than the ones carrying the marks of flour paste all through the leaves.

---

THE ROSE.—There is no more satisfactory plant than the Rose, and none more easily cared for. An annual enriching of the soil, winter protection where needed, destruction of injurious insects, and proper pruning constitute the essentials of its cultivation. In the first place, if the plants are provided with plenty of fertilizing material in a form that they can take up and assimilate when they commence to make their first growth in the spring they will, by their increased vigor, be better able to sustain themselves against mildew or insects ; thus enriching of the soil secures at the same time the ability to produce more and better flowers, and to contend against parasitic adversaries. Well rotted stable manure or the commercial phosphates will supply the needed nutriment. A garden syringe, and some whale oil soap with which to make a solution in water, are pretty much all that are needed to fight insects with ; the soap water will destroy the green fly and the slug, and keep at bay the little rose-hopper or thrips, and while used principally against these insects it will impede the work of some others. If the leaf-roller should appear to be active about the time the buds are filling, it can be crushed between the leaves, looking the bushes over carefully for this purpose. The correct pruning of Roses must be based on the fact that the flowers are produced on the new wood. To keep up a supply of new wood is the point to be aimed at. The new shoots grow from those which were produced last and have become hard and ripened, as it is technically called. The spring pruning should be deferred until the most severe weather is past, but it should be done while the buds are yet dormant. The skill to decide how much wood to remove can be acquired only by experience. A strong, vigorous plant can be allowed more proportionately than a weaker one. If a few large blooms are wanted, prune short, but for the greatest quantity, prune long. To get any good results from Climbing Roses pruning must be attended to systematically every year, and it is for want of this attention that they so speedily become ill-looking. With proper pruning most beautiful effects can be produced with them on walls, pillars or trellises.— *Vick's Magazine for June.*

### THE TWO TYPES OF GIRLS.

Take an English girl and put her beside an American girl whose ancestry is pure English and there is a remarkable difference between them in shape, nature and color. The American as a rule, is slender, fairer and slighter limbed, thinner featured and more vivacious and excitable in manner. The English girl is fuller, rosier in color, heavier in build, and calmer. The voice of the American is thin and high, that of the English girl is rich and low. But where you will find the greatest physical difference is in the feet and hands. The American's foot is small, thin, high arched and tendonous in the ankle. The English girl's is plump, fat and full in the ankle. There is the same difference in the hands. Take a cast from an English and American foot and any one can distinguish them with half an eye, all the attachments as they are called, are longer and more tendonous in the American than in the English.

Certainly there is a great difference in the general appearance of the English girl and the American. There is something charming in the one as of a rose, and in the other of a lily. Where the English have the advantage over the Americans is in their voices and intonations. An English woman's voice is a pleasure to hear —so sweet and low, and pleasant in its modulations—while the Americans whine with a high pitched voice. I wish they would correct this. You know them, "as the blind man knew the cuckoo, by the bad voice." They sing better than the English, because the English can never fully utter their voice and throw it out. The American girls are sometimes very handsome, and they generally have a refinement of look and feature, if not of manner. In their ways too, there is a certain wild willfulness and independence which, when it does not go too far (as it frequently does), is very attractive.—*Ex.*

---

### THE CANCER HOSPITAL.

The fifth annual report of the New York Cancer Hospital for the second year of the new hospital building, shows a gratifying amount of work done towards the relief of cancer patients. Two hundred and forty-one patients were admitted, 131 being free. One hundred and eighty-three operations were performed. Of the deaths 69 per cent. occurred in the case of patients who were incurable when admitted, and consequently were not operated upon.

The report emphasizes the fact that the institution is a cancer hospital, and that cases of malignant disease, however far advanced, receive the preference, as was the original intention of the founders. It is entirely dependent upon the public for financial support as far as the current expenses are concerned, and its doors stand open to all sufferers from cancer, whether they are able to pay for their board or not.

The opinion that the cancer germ is derived (like those of consumption, scarlet fever, etc.) from food animals, through indiscriminate flesh eating, vaccination, etc., derives support by the extensive observations of MM. Verneuil and Reclus, respecting the prevalence of cancer ; who find that persons who subsist upon a vegetable diet are much less subject to the disease than those who use flesh largely.

